# Gossypiboma 19 years after laminectomy mimicking a malignant spinal tumour: a case report

**DOI:** 10.1186/1752-1947-8-311

**Published:** 2014-09-18

**Authors:** Takashi Kobayashi, Naohisa Miyakoshi, Eiji Abe, Toshiki Abe, Tetsuya Suzuki, Masato Takahashi, Yoichi Shimada

**Affiliations:** 1 Department of Orthopedic Surgery, Akita Kosei Medical Center, 1-1-1 Iijima-Nishifukuro, Akita 011-0948, Japan; 2 Department of Orthopedic Surgery, Akita University Graduate School of Medicine, 1-1-1 Hondo, Akita 010-8543, Japan; 3 Department of Orthopedic Surgery, Akita Red Cross Hospital, 222-1 Nawashirosawa, Kamikitatesaruta, Akita 010-1406, Japan; 4 Department of Diagnostic Pathology, Akita Kosei Medical Center, 1-1-1 Iijima-Nishifukuro, Akita 011-0948, Japan

**Keywords:** Foreign body, Gossypiboma, Laminectomy, Malignant spinal tumour, Retained surgical sponge, Spinal surgery

## Abstract

**Introduction:**

Gossypiboma is rare and mostly asymptomatic in chronic cases. It can be confused with other soft tissue masses.

**Case presentation:**

Our patient was an 87-year-old Japanese man with a history of surgery for a lumbar lesion causing lumbar canal stenosis 19 years earlier. Computed tomography showed a soft tissue mass with osteolysis and periosteal thickening of the vertebral lamina. On magnetic resonance imaging, the mass showed heterogeneous signal intensity on T2-weighted imaging, suggesting a malignancy. At the time of biopsy, small pieces of retained surgical sponge were collected. Surgical treatment was performed to excise the soft tissue tumour.

**Conclusions:**

Gossypiboma should be included in the differential diagnosis of soft tissue masses in the paraspinal region in patients with a history of previous spinal surgery.

## Introduction

Surgical sponges with radiopaque markers are easily recognized on plain radiographs, but retained foreign bodies without such markers present a difficult diagnostic problem. This intraoperative complication has rarely been reported in the paraspinal area [1-10]. Gossypiboma is a term used to describe a mass within the body that consists of a cotton matrix surrounded by a foreign body reaction. A patient in whom a gossypiboma was discovered beside a vertebral lamina 19 years after lumbar surgery is described, and the radiological appearance and differential diagnosis are reviewed.

## Case presentation

**Figure 1:**
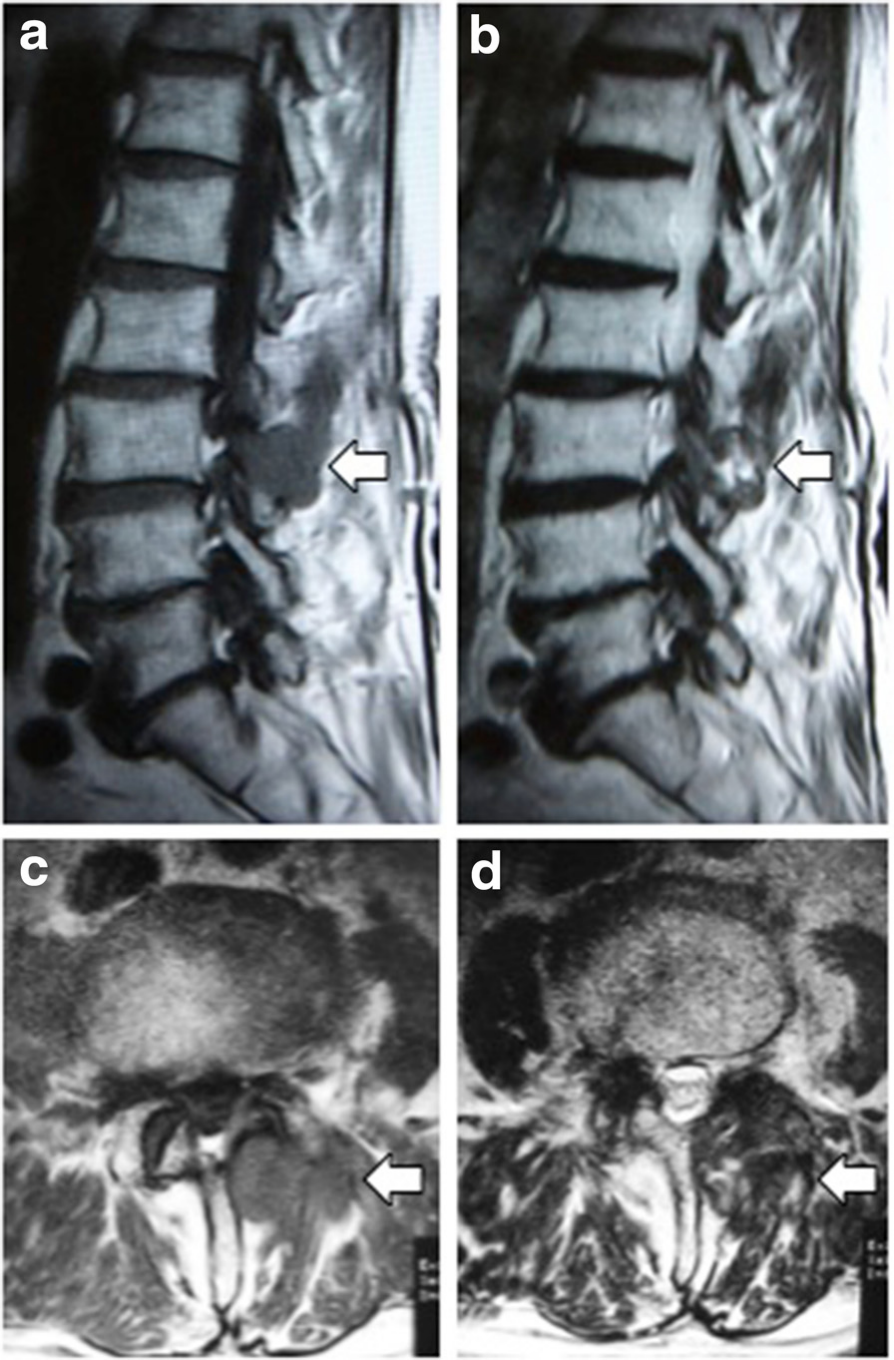
**Magnetic resonance imaging sagittal and axial images.** A soft tissue mass (open arrow) at the L3 to 4 level is hypointense compared with the spinal cord on the T1-weighted image **(a, c)**, and heterogeneous signal intensity is seen on the T2-weighted image **(b, d)**.

**Figure 2:**
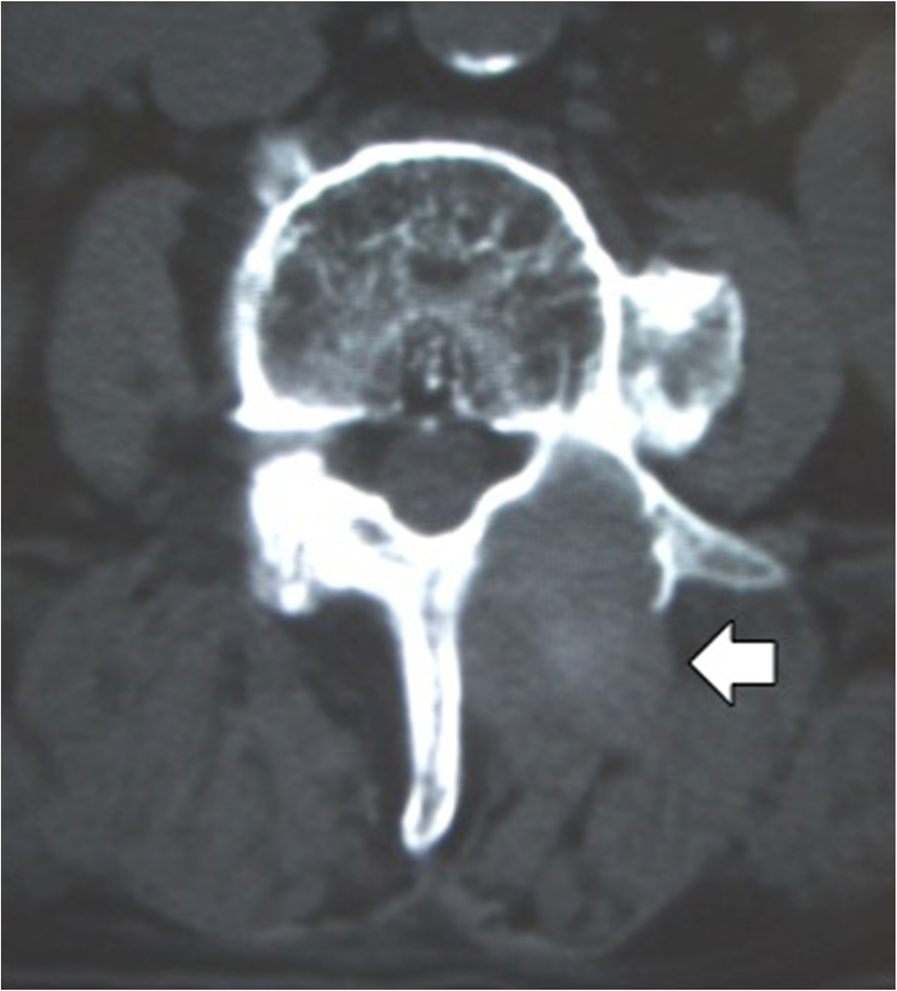
**Computed tomography axial image.** A mass (open arrow) is seen posterior to the lamina with osteolytic change.

**Figure 3:**
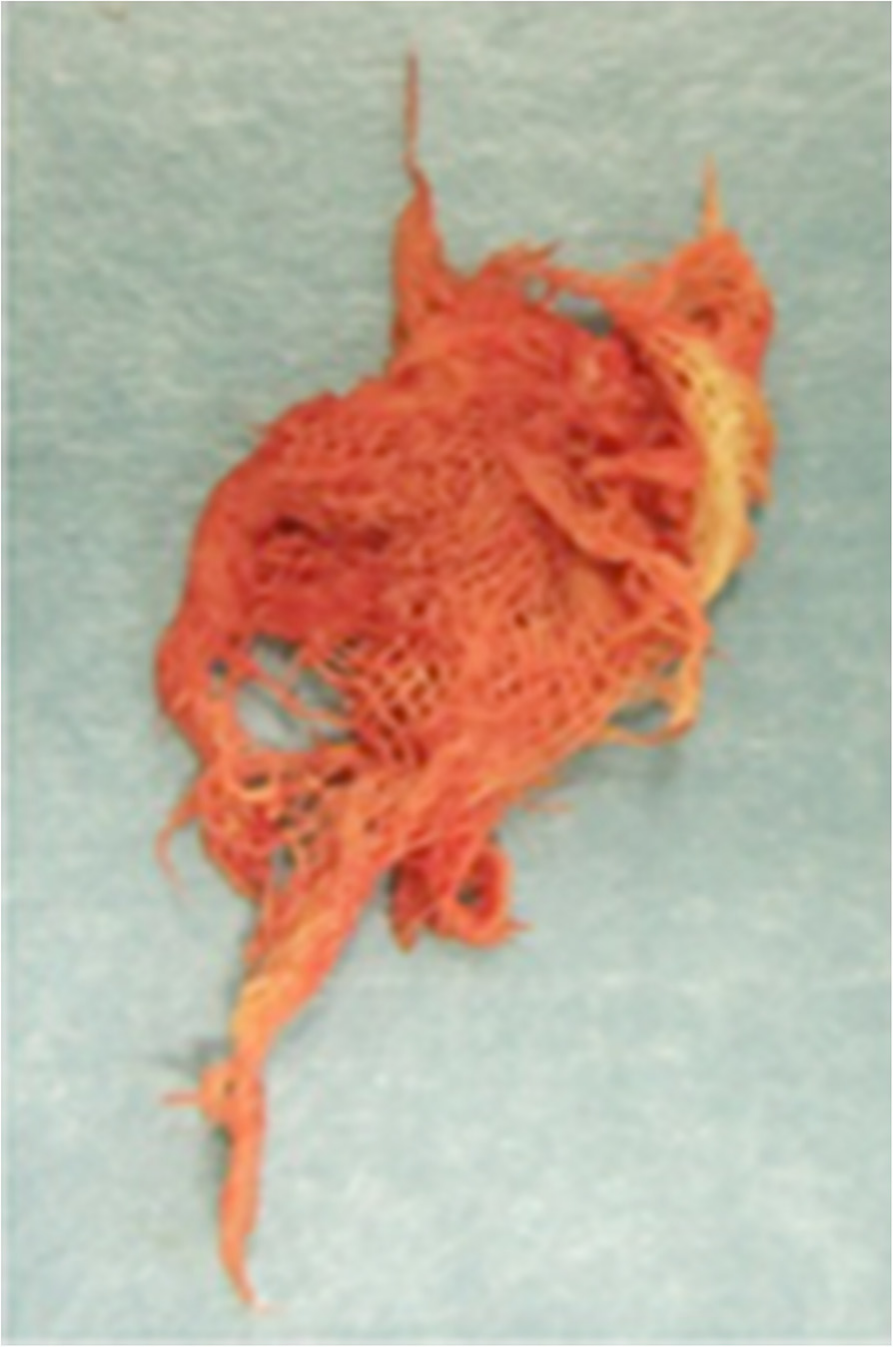
Macroscopic appearance of the retained sponge material at biopsy.

**Figure 4:**
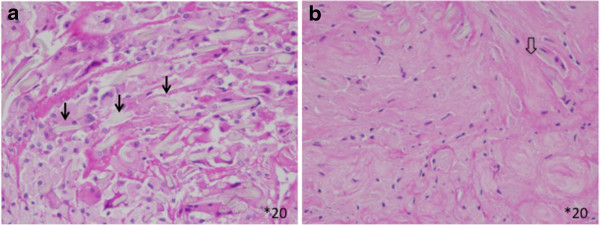
**Histological findings.** A fresh foreign body reaction around the gauze fibre (arrow, **a**) and fibrin deposition (open arrow), which indicates an old foreign body reaction **(b)** are seen (hematoxylin and eosin ×20).

An 87-year-old Japanese man was admitted to our hospital because on an earlier visit to another hospital for his lumbago he was found to have a paraspinal mass on magnetic resonance imaging (MRI). He had undergone surgery for lumbar canal stenosis 19 years earlier at another hospital. At our out-patient clinic, his temperature was 37.1°C, blood examinations showed leucocytosis (white blood cell; 8700/μL, normal range 4000 to 8000/μL) and increased C-reactive protein (0.9mg/dL; normal range 0 to 0.2mg/dL). The neurological examination was normal. MRI revealed a soft tissue mass at the L3 to 4 level. The mass lesion was hypointense compared with the spinal cord on T1-weighted imaging, with heterogeneous signal intensity on T2-weighted imaging (Figure 1). Plain computed tomography (CT) showed a mass posterior to the lamina with osteolytic change (Figure 2). A malignant tumour was suspected, and a biopsy was performed. At the time of biopsy under local anaesthesia, small pieces of gauze material were removed (Figure 3), and gossypiboma was diagnosed. The soft tissue mass was excised under general anaesthesia. During the operation, a retained surgical sponge was found on the lamina and excised completely with the fibrous capsule surrounding it. The lamina of L3 was concave and filled with granulation tissue. Its surface was very hard. The cavity was irrigated with saline, and the layers were closed anatomically. The histopathological assessment was reported as granulation due to the foreign body (Figure 4). Cultures taken at surgery were sterile. The postoperative period was uneventful, and the patient was in good condition 1 year after the operation.

## Discussion

Gossypiboma after spinal surgery has been rarely reported and occurs much less commonly than after operations in the abdominal cavity [11]. Kaiser *et al*. reported that, of the 9729 closed claims reviewed, 40 patients had retained surgical sponges, and two of 40 patients were post-laminectomy [11].

Gossypiboma can remain asymptomatic for many years and is mostly detected incidentally by radiologic investigations. Since cotton sponges are inert, they do not stimulate specific biochemical reactions without granuloma formation [1-10].

Various imaging techniques are used to diagnose gossypiboma. If the sponge has a radiopaque marker, the diagnosis can be made easily by X-ray examination. On CT, these lesions appear as well-circumscribed masses that contain hyperdense material and show capsular enhancement in the abdomen [12,13]. In the lumbar region, bone erosion or a bone cavity has been reported [1,8,9]. These osteolytic changes with marginal sclerosis may be evidence of the benign nature of the lesion and may be specific for gossypiboma around the lamina. MRI usually shows a well-defined mass with a fibrous capsule that exhibits low-signal intensity on T1-weighted imaging and high-signal intensity on T2-weighted imaging within the centre of the lesion and low-signal intensity mimicking a paraspinal abscess [2,4-7,9,10]. Only three cases were heterogeneous on T2-weighted imaging [1,3,8]. In the present case, heterogeneous signal intensity on T2-weighted imaging led to suspicion of a malignant tumour. At operation, granular tissue ingrowth into the surgical sponge was observed. On histological examination, a fresh foreign body reaction around the gauze fibre and fibrin deposition, which indicates an old foreign body reaction, was found. These histological findings may be related to the heterogeneous intensity on MRI T2-weighted imaging.

The diagnosis of gossypiboma is sometimes very difficult. Although chronic gossypiboma may be inert, sponges with radiopaque markers should be used to identify retained surgical sponges as soon as possible.

## Conclusions

Retained surgical sponges do not show specific clinical and radiological signs. They should be included in the differential diagnosis of soft tissue masses in the paraspinal region in patients with a history of previous spinal surgery.

## Consent

Written informed consent was obtained from the patient for publication of this case report and accompanying images. A copy of the written consent is available for review by the Editor-in-Chief of this journal.

## Abbreviations

CT: Computed tomography; MRI: Magnetic resonance imaging.

## Competing interests

The authors declare that they have no competing interests.

## Authors’ contributions

Surgery was performed by TK, TS, and TA. TK, NM, EA, and MT were the major contributors in writing the manuscript. EA and YS supervised the whole work. All authors read and approved the final manuscript.

## References

[B1] Aydogan M Mirzanli C Ganiyusufoglu K Tezer M Ozturk I A 13-year-old textiloma (gossypiboma) after discectomy for lumbar disc herniation: a case report and review of the literature Spine J 2007 7 618 621 10.1016/j.spinee.2006.08.004 17905325

[B2] Atabey C Turgut M Ilica AT Retained surgical sponge in differential diagnosis of paraspinal soft-tissue mass after posterior spinal surgery: report of eight cases Neurol India 2009 57 320 323 10.4103/0028-3886.53289 19587475

[B3] Hakan T Aydoseli A Demir K Aker F Clinical, pathological and radiological features of paraspinal textiloma: report of two cases and review of the literature Neurol Neurochir Pol 2009 43 475 478 20054750

[B4] Is M Karatas A Akgul M Yildirim U Gezen F A retained surgical sponge (gossypiboma) mimicking a paraspinal abscess Br J Neurosurg 2007 21 307 308 10.1080/02688690701367701 17612926

[B5] Kim HS Chung T-S Suh SH Kim SY MR imaging findings of paravertebral gossypiboma AJNR Am J Neuroradiol 2007 28 709 713 17416826 PMC7977336

[B6] Kucukyuruk B Biceroglu H Abuzayed B Ulu MO Kafadar AM Paraspinal gossybipoma: A case report and review of the literature J Neurosci Rural Pract 2010 1 102 104 10.4103/0976-3147.71725 21808514 PMC3139335

[B7] Okten AI Adam M Gezercan Y Textiloma: a case of foreign body mimicking a spinal mass Eur Spine J 2006 15 626 629 10.1007/s00586-006-0136-6 16736201 PMC1602194

[B8] Rohde V Küker W Gilsbach JM Foreign body granuloma mimicking a benign intraspinal tumour Br J Neurosurg 1999 13 417 419 10.1080/02688699943574 10616573

[B9] Stoll A Retained surgical sponge 40 years after laminectomy Case report. Surg Neurol 1988 30 235 236 10.1016/0090-3019(88)90278-9 3413671

[B10] Turgut M Akyüz O Ozsunar Y Kacar F Sponge-induced granuloma (“gauzoma”) as a complication of posterior lumbar surgery Neurol Med Chir (Tokyo) 2005 45 209 211 10.2176/nmc.45.209 15849460

[B11] Kaiser CW Friedman S Spurling KP Slowick T Kaiser HA The retained surgical sponge Ann Surg 1996 224 79 84 10.1097/00000658-199607000-00012 8678622 PMC1235250

[B12] Choi BI Kim SH Yu ES Chung HS Han MC Kim CW Retained surgical sponge: diagnosis with CT and sonography AJR Am J Roentgenol 1988 150 1047 1050 10.2214/ajr.150.5.1047 3282401

[B13] Dewachter P Van De Winkel N Retained surgical sponge JBR-BTR 2011 94 118 119 10.5334/jbr-btr.531 21874803

